# Antioxidant and Anti-inflammatory Effect of Nrf2 Inducer Dimethyl Fumarate in Neurodegenerative Diseases

**DOI:** 10.3390/antiox9070630

**Published:** 2020-07-17

**Authors:** Sarah A. Scuderi, Alessio Ardizzone, Irene Paterniti, Emanuela Esposito, Michela Campolo

**Affiliations:** Department of Chemical, Biological, Pharmaceutical and Environmental Sciences, University of Messina, Viale Ferdinando Stagno D’Alcontres, 31-98166 Messina, Italy; sarascud@outlook.it (S.A.S.); aleardizzone@unime.it (A.A.); ipaterniti@unime.it (I.P.); campolom@unime.it (M.C.)

**Keywords:** dimethyl fumarate (DMF), oxidative stress (OS), Alzheimer’s disease (AD), Parkinson’s disease (PD), Huntington’s disease (HD), amyotrophic lateral sclerosis (ALS)

## Abstract

Neurodegenerative diseases (NDs) represents debilitating conditions characterized by degeneration of neuronal cells in specific brain areas, causing disability and death in patients. In the pathophysiology of NDs, oxidative stress, apoptosis and neuroinflammation have a key role, as demonstrated by in vivo and in vitro models. Therefore, the use of molecules with antioxidant and anti-inflammatory activities represents a possible strategy for the treatment of NDs. Many studies demonstrated the beneficial effects of fumaric acid esters (FAEs) to counteract neuroinflammation and oxidative stress. Among these molecules, dimethyl fumarate (DMF) showed a valid therapeutic approach to slow down neurodegeneration and relieve symptoms in patients with NDs. DMF is a methyl ester of fumaric acid and acts as modulator of the nuclear factor erythroid 2-related factor 2 (Nrf2) pathway as well as nuclear factor kappa-light-chain-enhancer of activated B cells (NF-κB) translocation. Therefore, this review aims to examine the potential beneficial effects of DMF to counteract oxidative stress and inflammation in patients with NDs.

## 1. Introduction

Neurodegenerative diseases (NDs) are debilitating conditions caused by the progressive degeneration and death of neurons; the prevalence of these diseases is rising in today’s society [1]. NDs include a large spectrum of heterogeneous and multifactorial pathologies as Alzheimer’s disease (AD), Parkinson’s disease (PD), Huntington’s disease (HD), and amyotrophic lateral sclerosis (ALS).

**Figure 1:**
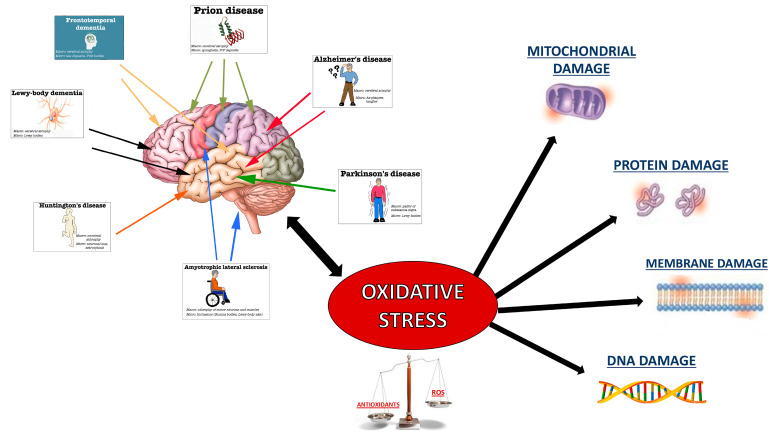
Interconnection between OS and NDs. The figure shows the main NDs (on the left; colored arrows represent the specific region for every NDs), while on the right, the damage emerging by OS—the common point for all NDs.

Although some genetic factors have been identified, the pathophysiology of NDs remains poorly understood [2]. NDs are specifically characterized by apoptosis and/or necrosis accompanied by dysfunction of neuronal cells. The brain, for the high oxygen consumption and lipid-rich content, is highly susceptible to oxidative stress (OS) [3]; in fact, many studies underlined a significant connection between OS and NDs. OS is generally caused by an imbalance between oxidant species and antioxidant defense systems as summarized in Figure 1.

The imbalance occurs as a result of an overproduction of reactive oxygen species (ROS), reactive nitrogen species (RNS), or improper functioning of the antioxidant system [4]. OS has an important role in the pathogenesis of NDs, often associated with specific protein clusters such as α-synuclein, DJ-1, β-amyloid, tau protein and/or signaling pathways [5], which cause the deterioration of neuronal cells, the formation of neurofibrillary tangles and senile plaques.

The molecular mechanisms of OS include inflammation, mitochondrial dysfunction, and apoptosis that culminates in neuronal death [6]. In NDs, inflammation is characterized by activation of microglia and astrocytes in the brain parenchyma, which lead to the production of inflammatory mediators, ROS and RNS [7], that including superoxide radical anion (O_2_^•−^), hydrogen peroxide (H_2_O_2_), hydroxyl radical (^•^OH), nitric oxide (NO), and peroxynitrite (ONOO^−^).

Moreover, it is interesting to highlight how astrocytes would seem to play an important role on a cellular level, particularly in the case of ALS and PD. It should be considered that ARE-regulated Nrf2-dependent genes are also preferentially activated in astrocytes, thus providing an interesting cellular and molecular link between astrocyte dysfunction and the role of oxidative stress in neurodegeneration [8]. In particular, Nrf2 has been proposed as a new pharmacological target in neuroinflammatory pathologies, including NDs [9]. Many papers highlighted the importance of molecules with antioxidant activities for the treatment of NDs, such as the fumaric acid esters (FAEs), where DMF represents the most pharmacologically effective molecule [10]. It has been shown that DMF acts as an Nrf2 activator, able to stimulate a cellular defense to protect neurons from ROS-induced damage [11]. Therefore, this review aims to highlight the involvement of OS in neurodegenerative diseases and the effect of DMF in the treatment of these diseases.

## 2. Neurodegenerative Diseases Background

### 2.1. Alzheimer’s Disease

AD is a chronic and progressive neurodegenerative disorder [12]; it is the most common type of dementia that affects more than 46.8 million people worldwide, especially in older people [13]. AD is a heterogeneous and multifactorial neurodegenerative disease [14]; initially, AD is characterized by a sporadic decline in episodic memory, followed by a more global decline in cognitive abilities, such as long-term loss of memory, language, attention, and personality changes [15].

The majority of AD cases are sporadic, caused by genetic and environmental factors that contribute to the genesis of the disease [16,17]. Whereas, other cases of AD are due to mutations in specific genes such as amyloid-β protein precursor (APP), presenilin 1 and presenilin 2 [18].

The pathophysiology of AD is characterized by an extraneuronal deposition of the β- amyloid protein (Aβ) in the form of plaques and by an intraneuronal aggregation of the microtubule-associated protein tau in specific brain regions such as the hippocampus and cerebral cortex [19].

In AD, the deposition of Aβ causes a chronic inflammatory response which contributes to neurodegeneration [14]. The mechanisms and pathways of neuroinflammation triggered by Aβ protein have been shown in vivo and in vitro [20,21]. In AD, the mechanism of inflammation is mediated by activation of microglia and astrocytes, followed by the production of cytokines and chemokines [22].

In response to Aβ-induced damage, astrocytes showed a significant increase in the expression of the glial fibrillary acidic protein (GFAP) and vimentin (VIM), markers of astrogliosis [23].

Moreover, activated astrocytes induce the nuclear translocation of *nuclear factor kappa-light-chain-enhancer of activated B cells* (NF-κB) [21], promoting the release of pro-inflammatory cytokines such as interleukin-1 (IL-1), IL-6, tumor necrosis factor-α (TNF-α) and chemokines [21]. In response to Aβ, chronically activated microglia release pro-inflammatory and toxic products that include ROS, nitric oxide (NO), IL-1β and IL-6, which in turn stimulates the activation of CDK5, a kinase known to hyperphosphorylate tau protein [24].

Currently, no effective disease-modifying therapies are available in clinical practice. However, some treatments have been approved for the symptomatic management of AD and consist mostly of acetylcholinesterase inhibitors (AChE) and N-methyl-D-aspartate (NMDA) receptor antagonists to alleviate the cognitive and functional deficits, even if only for a limited time [25].

### 2.2. Parkinson’s Disease

PD is the most common pathology characterized by motor disorders and the second-most common neurodegenerative disease after AD [26]. It is estimated that it affects about 5 million people every year, with a higher incidence in males than in females; the average age of onset is around 60 years where the prevalence and incidence increase exponentially with age [27]. In the early stages of the disease, symptoms are presented that relate to movement, including tremors, stiffness, bradykinesia, and difficulty in walking; subsequently, cognitive and behavioral problems can arise, with dementia in the advanced stages [28]. The symptomatology has its basis in the etiopathogenesis of PD; it is known that PD has multifactorial origins, which include genetic and environmental factors [29,30,31,32,33]. Both genetic and environmental factors cause degeneration of the neurons of the substantia nigra pars compacta [34]; this degeneration is due to an alteration of the closed-circuit acetylcholine-GABA-dopamine that reduce dopamine content and increase the level of acetylcholine at the neuronal level [35,36,37,38,39]. In fact, the treatment for Parkinsonian patients acts to attenuate the symptoms and can be used both in the early and in the advanced stages of the disease.

The common therapy involved are the administration of Levodopa, a dopamine precursor, cholinergic antagonists, and monoamine oxidase B (MAO-B) inhibitors. [40,41,42]. However, drug therapy still cannot completely cure the disease; therefore, it is of fundamental importance to deepening the neurological molecular mechanisms that underlie the development of the disease; in fact, the neurodegenerative aspects are closely related to the immune system, neuroinflammation and oxidative stress [43,44]. Therefore, in-depth knowledge of these multifactorial aspects could be helpful in the discovery of new therapeutic targets for the management of PD.

### 2.3. Huntington’s Disease (HD)

HD is a neurodegenerative disorder characterized by progressive motor, cognitive, and psychiatric symptoms [45]. HD is caused by an abnormal extension in the cytosine–adenine–guanine (CAG) repeat in the huntingtin (HTT) gene, causing the mutation of the huntingtin protein [46], which is generally involved in the development of the nervous system.

HD is characterized by progressive degeneration and death of striatal neurons in CNS. In HD patients, symptoms progress over 15–20 years; motor functions are affected initially, while cognitive impairment and dementia can be observed at later stages of the disease [45].

Moreover, elevated concentrations of heme oxygenase 1 (HO-1) 3 -nitrotyrosine and MDA have been detected in HD patients post mortem, particularly in the cerebrospinal fluid, in the striatum, and the cortex [47]. Many studies suggest that mHTT promotes the abnormal release of cytokines by activated microglia [48,49]. HD patients have been shown high levels of proinflammatory cytokines, including IL-1β, IL-6, and TNFα, in their plasma and striatal tissues, and promotes the activation of the nuclear factor (NF)-κB-p65 [50].

### 2.4. Amyotrophic Lateral Sclerosis (ALS)

Among the various NDs, ALS is the most common type of motor neuron disease. ALS is a neurodegenerative disorder characterized by the progressive degeneration of upper and lower motor neurons in the spinal cord, cortex, and brainstem [51].

ALS is characterized by OS and neuroinflammation. In ALS, neuroinflammation is mediated by astrocytes and microglia activation [52,53]. The reactive microglia release inflammatory cytokines as which high levels have been found in cerebrospinal fluid (CSF) and blood from ALS patients.

Recently, 24 additional genes have been connected with sALS and fALS [54] including p62 [55], which has a direct link to Keap1/Nrf2 and autophagy. Nrf2 and Keap1 have been analyzed in the primary motor cortex and spinal cord of ALS patients [56]. These studies have shown a reduction in Nrf2 mRNA in ALS patients tissues relative to Nrf2 levels in control tissues, suggesting that alterations in this signaling cascade may contribute to motor neuron degeneration in ALS.

## 3. Involvement of OS in NDs

### 3.1. Alzheimer’s Disease

In the development of AD, OS plays a central role. In AD, OS promotes Aβ deposition and the loss of synapses and neuronal cells [57]. In specific brain regions of AD patients, like the hippocampus, temporal cortex, and amygdala, a significant increase has been shown in malondialdehyde (MDA) and acrolein levels, markers for lipid peroxidation [58,59,60], and an increase of 3-nitrotyrosine level, a marker for peroxynitrite-mediated protein nitration [61].

Moreover, different studies suggested that increased levels of Aβ in the brain of AD patients could accelerate ROS production by binding to mitochondrial membranes, altering mitochondrial function [62]. Dysfunction in mitochondrial function contributes to dysregulation of neuronal calcium homeostasis, causing synaptic dysfunction and neurodegeneration [63,64]. Furthermore, many papers showed a decrease of Nrf2 activity in AD on hippocampal and cortex neurons [65,66].

### 3.2. Parkinson’s Disease

Oxidative stress is one of main factors leading to cell dysfunction and cell death in PD [67,68,69], thus causing mitochondrial damage and neuroinflammation [70]. The oxidative condition in PD is due to both exogenous [71] (environmental toxins, ionizing radiation, UV rays, smoke) and endogenous factors [72] like free radicals. Free radicals produced following the catabolism of catecholamines, including dopamine [73], represents the link between OS and PD. Specifically, the damage of the neurons of the substantia nigra originate in the hyperproduction of free radicals during the oxidative metabolism (autoxidation) of dopamine [73]; in particular, this increase in dopaminergic metabolism, [74] together with the depletion of reduced glutathione (GSH) [75] and a large presence of Fe^2+^ ions [76], favor an overproduction of free radicals, which contributes to an increase in the aggregation of synuclein and other misfolded proteins [73]. Recent articles highlighted the roles of two proteins, c-Rel and Leucine-rich repeat kinase (LLRK) 2, which would seem to have a crucial role in OS related to PD. The c-Rel protein is a transcriptional regulator for mitochondrial antioxidant and antiapoptotic factors, therefore, the reduction of its physiological production could contribute to OS in PD [77]; while mutations of the LLRK2 enzyme in PD would seem related to mitochondrial ROS production, thus suggesting the involvement of this enzyme in the regulation of OS and mitochondrial dysfunction [78].

### 3.3. Huntington’s Disease (HD)

OS and neuroinflammation are among the common pathogenic mechanisms implicated in HD [47,79,80]. Different studies demonstrated that the accumulation of mutant huntingtin (mHtt) causes an alteration of mitochondrial calcium buffering and reduces the expression of the peroxisome proliferator-activated receptor-γ (PPARγ) and coactivator-1 α (PGC-1α), a transcriptional coactivator involved in the mitochondrial respiration and ROS metabolism [81].

Various therapies have been adapted to counteract OS and inflammation in HD; in particular, the stimulation of the Nrf-2/ARE pathway has be proven to be efficacious, associated with increased expression of antioxidant enzymes and reduction of ROS levels in CNS [82].

### 3.4. Amyotrophic Lateral Sclerosis (ALS)

The etiology of the disease is still unknown, but it was found that approximately 10% of familial ALS (FALS) cases are the result of a mutation in the gene responsible for the SOD-1 encoding, whereas 90% of ALS cases are sporadic (sALS) and it is not clear the genetic connection [51]. SOD-1 is an antioxidant enzyme covering a key role in the regulation of OS [83]. Therefore, mutations of SOD-1 predispose cellular organelles to oxidative damage causing elevate ROS production.

The reactive microglia release inflammatory cytokines as which high levels have been found in cerebrospinal fluid (CSF) and blood from ALS patients. On the other hand, mitochondrial dysfunction causes excessive ROS production, which has been linked to neurodegeneration in ALS.

Furthermore, a substantial number of clinical studies have demonstrated that levels of MDA and 8-hydroxyguanosine (8-OHdG), a marker of oxidative DNA damage, were significantly increased in the peripheral blood of ALS patients [84,85].

Therefore, Nrf2-ARE pathway activation could be a valid therapeutic target for the treatment of neurodegeneration and OS in ALS patients [86].

## 4. Dimethyl Fumarate

### 4.1. DMF Structure and Origin

FAEs, also known as fumarate, are derivatives of fumaric acid which occurs naturally in many fruits and vegetables. It has been isolated for the first time from the roots of the wild herbaceous plant *Fumaria officinalis* (Fumariaceae). Anti-inflammatory and antioxidant activities have been attributed to this class of molecules, among which DMF represents the pharmacologically most effective molecule among FAEs [87]. Chemically, DMF is the methyl ester of fumaric acid. In human, DMF is extensively metabolized by esterase before reaching systemic circulation and further metabolism occurs through the tricarboxylic acid cycle (Krebs cycle and citric acid cycle), without any involvement of the cytochrome P450 system [11].

### 4.2. DMF Metabolism and Function

**Figure 2:**
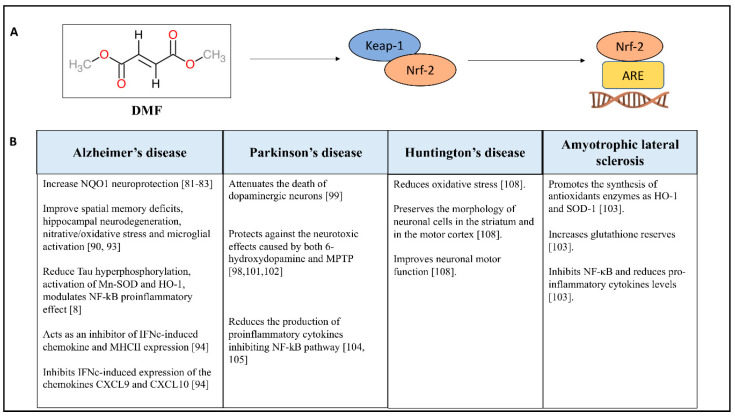
Properties of DMF in NDs. (**A**) shows the mechanism of action of DMF through Keap1/Nrf2/ARE pathway; (**B**) provides a table which summarizes the main effects of DMF in NDs (specifically for AD, PD, HD, ALS).

DMF is considered a prodrug because after administration, usually oral, DMF is cleaved into monomethyl fumarate (MMF) and fumarate via esterase inside cells into the small intestine DMF [88] and MMF have half-lives of 12 min and 36 h, respectively. The maximum concentration of the MMF is reached within 5–6 h [89]. FAEs have been used for years as a therapy for psoriasis, a chronic inflammatory skin disease mediated by skin-directed T cells, resulting in scaly plaques [90,91,92]. Clinical studies reported high efficacy, thanks to its immunomodulatory activity also in the treatment of MS [93,94,95]. Thanks to the results obtained from clinical studies, in 2013 DMF was licensed as an oral therapeutic agent for the treatment of relapsing forms of multiple sclerosis [96,97]. Furthermore, it has been disclosed that DMF and MMF act on Kelch-like ECH associating protein 1 (Keap1), an Nrf2 activator (Figure 2A), which works on both antioxidant and inflammatory pathways [98], promoting the attenuation of pro-inflammatory cytokine production [99] and the modulation of microglia and astrocytes [100].

### 4.3. Nrf2 Structure and Function

Nrf2 is ubiquitously expressed in the body, including in the central nervous system (CNS) [101]. Nrf2 regulates and induces the expression of cytoprotective antixenobiotic and antioxidant enzymes [102]. However, Nrf2 activity declines with age, which is the main risk factor for Parkinson’s and Alzheimer’s [103]. Under physiological conditions, Nrf2 is bound in the cytoplasm to Keap1; upon exposure to ROS or xenobiotics, Nrf2 translocates into the nucleus, then heterodimerizes and binds to the antioxidant response element (ARE) promoter region. The binding of Nrf2 to ARE induces the transduction of antioxidant enzymes, such as glutathione-S-transferase and HO-1 [104]. It is identified that Nrf2 activity is linked to the NF-kB pathway, which is more readily activated in oxidative environments [105]. Therefore, considering the cross-talk that exists between NF-kB and Nrf2 pathways provides DMF the ability to counteract the inflammation. Given these considerations, the use of DMF could improve the management of many pathologies including the NDs (Figure 2B); this thesis is supported by different research articles that demonstrate the efficacy of DMF in many in vitro and in vivo models of NDs.

## 5. DMF and NDs

### 5.1. DMF and Alzheimer

The studies that correlate DMF to AD are few; in fact, this research field is still in continuous evolution. The first studies conducted between the late 90s and early 2000s demonstrated the ability of DMF to activate endogenous antioxidant defenses in neuronal cells that could counteract the damage produced by free radicals [106], in particular, increasing the production of the enzyme NAD(P)H quinone oxidoreductase 1 (NQO1), which exerts a neuroprotective function in hippocampal pyramidal neurons in AD patients [107,108]. NQO-1 belongs to NAD(P)H dehydrogenase (quinone) family—these species encode for a cytoplasmic 2-electron reductase that reduces quinones to hydroquinones. The enzymatic activity of this target gene Nrf2 prevents the reduction of the enzymatic levels of quinones (which would cause the production of radical species) and thus exerts antioxidant effects [8]. Some studies identified p62 as activator Nrf2 [109,110,111]; it has been hypothesized that p62 is phosphorylated in the AD brain and that the phosphorylated p62 binds to Keap1, with consequent accumulation of Nrf2 [101]. In addition, with the phosphorylation of p62, a decrease in the cytosolic expression of Keap1 and an increase in the expression of the target gene Nrf2 in the brain were observed [66].

Other studies conducted by Majkutewicz et al. [112,113] analyzed the effect of DMF on spatial memory impairment and hippocampal neurodegeneration on the therapeutic effect of DMF in the streptozotocin (STZ)-induced model of sporadic AD. The data showed that DMF improved spatial memory deficits, hippocampal neurodegeneration, nitrative/oxidative stress, and microglial activation caused by an intracerebroventricular (ICV) injection of STZ [112].

Moreover, an in vitro study investigated the multiple mechanisms of DMF [10]; for the experiment, were used SH-SY5Y human neuroblastoma cell lines stimulated with amyloid-beta (Ab). The form of 42-amino acid of this peptide, Ab (1–42), has been shown to contribute to the pathogenesis of AD, and is also linked to oxidative stress and inflammation in the CNS [114].

The study, performed by Campolo et al., indicated that DMF operated its protective role by: 1. reduction in Tau hyper-phosphorylation; 2. Reduction of the Nf-kB pro-inflammatory pathway; and 3. Modulation of the Nrf2 pathway increasing antioxidants enzymes production; these results confirmed the involvement of Nrf2 and NFkB systems to protect against cytotoxicity induced by β-amyloid and promoting DMF as a potential treatment for limiting the inflammatory and oxidative stress in neurons following amyloid peptides formation [10].

Furthermore, following a systemic immune challenge, DMF treatment was able to reduce acute microglial activation, neuroinflammation, long-term memory deficits, and responses of reactive astrocytes [115], thus adding also an immune hypothesis in the multifactorial causes of AD.

It is interesting to highlight a study by Rojo et al. [116] that used a new mouse model of AD that combines amyloidopathy and tauopathy. The study proved that low-grade chronic neuroinflammation is a “primer” of neurodegeneration, and not just a result, therefore an antioxidant modulator of inflammation as DMF, which targets NRF2, protected from AD disease progression [116].

### 5.2. DMF and Parkinson

The first studies evaluating the efficacy of DMF in PD are relatively recent. Some promising results have been obtained by studies on Nrf2 knock-out mice, in which the lack of this transcription factor caused a greater loss of dopaminergic afferents in the striatum. However, it has been shown how the overexpression of Nrf2 in astrocytes protects against the damage caused by 6-hydroxydopamine in mice [117,118]. Similar results were also obtained by a study of Lastres-Becker et al. [119] in which Nrf2 activation attenuating the death of dopaminergic neurons in DMF-treated mice. Moreover Nrf2 deficiency is associated with an increase of 1-methyl-4-phenyl-1,2,3,6-tetrahydroxydopamine (MPTP) sensibility; on the other hand, astrocytic Nrf2 overexpression, in mice, protect from the toxic effect of MPTP [120]. So certainly Nrf2 activation improved the antioxidant response and protects against the neurotoxic effects caused by both 6-hydroxydopamine and MPTP, as confirmed in various in vivo models [120,121,122].

DMF was able to attenuate neurotoxicity in in vitro model of 6-OHDA-stimulated SH-SY5Y cells and also in an animal model of Parkinson’s disease by enhancing Nrf2 activity and reducing the production of proinflammatory cytokines in splenocytes of mice as reported by Jing et al. [123].

A study, including both an in vitro and in vivo models, was conducted by Campolo et al. [124]; the research article highlighted how DMF exerts neuroprotective action in an MPTP-mouse model of PD, this action is given not only by the DMF’s ability to regulate antioxidant enzymes such as Nrf2, Mn-SOD and HO-1, but also by the anti-inflammatory action deriving from the cross-talking between Nrf2 and NF-κB pathways, thus decreasing levels of pro-inflammatory cytokines like IL-1 and pro-inflammatory enzymes like cyclooxygenase (COX)-2 and Inducible nitric oxide synthase (iNOS). Besides, the results of this study underlined how DMF is capable of significantly reducing neuronal cell degeneration in the dopaminergic tract and behavioral impairments, preventing dopamine depletion, increasing tyrosine hydroxylase and dopamine transporter activities and also reducing the number of α-synuclein- positive neurons.

Therefore, the CNS-protective effects of DMF could involve regulation of Nrf2-mediated oxidative stress and inflammatory response mechanisms implicated in a variety of pathological conditions [124].

In addition, human data showed that Nrf2 dependent factors NQO1 and HO-1, are strongly up-regulated in glial cells in postmortem tissue from PD patients, possibly indicating a naturally occurring event that is insufficient to counteract the damage suggesting an endogenous approach strategy for PD [125,126]. Therefore, the neuroprotective property of DMF, in addition to its safety profile, may represent a helpful and complementary therapeutic approach that can be applied to future clinical practice in PD.

### 5.3. DMF and Huntington

Many papers demonstrated the neuroprotective effect of DMF in HD, thanks to its ability to activate the Nrf2 transcriptional pathway [127].

The therapeutic efficacy of DMF was investigated in a study performed by Ellrichmann et al. [127], on R6/2 and YAC128 transgenic mice models of HD. DMF treatment was able to preserve the morphology of neurons in the striatum and the motor cortex, exerting beneficial effects on survival and motor behavior in R6/2 mice [127]. Moreover, DMF treatment led to a significant reduction in dyskinesia as well as diminished neurodegeneration [128].

The efficacy of DMF as an inducer of the oxidative stress response in models of HD underlines the importance of free radicals in the pathophysiology of HD [127]. Furthermore, DMF thanks to its antioxidants effects could represent a valid approach therapeutic for HD treatment [128].

### 5.4. DMF and ALS

In the CNS regions of patients with ALS, high levels of ROS, and changes in antioxidant defense system have been revealed. Therefore, new therapies that improve cellular resistance to free radicals could prove useful for ALS treatment [129].

Many studies highlighted the key role of astrocyte on Nrf2 activation in ALS since activation of Nrf2 coordinates the up-regulation of antioxidant defenses systems such as SOD-1 [130,131]. Various molecules activating Nrf2 have been assessed in murine models of ALS; among these molecules, DMF [132]. A clinical trial conducted by Vucic et al. assessed the efficacy of DMF treatment in sporadic ALS, using a commercially available medication approved for the treatment of the relapsing-remitting form of multiple sclerosis [133]. In this study, DMF treatment showed to reduce pro-inflammatory T-cells levels including CD4^+^, interferon (IFN)γ, CD8^+^, induce Nrf2 activity in astrocytes and preserve the motor neurons function, also stabilizing respiratory function in patients [134].

Therefore, this study demonstrated how DMF treatment slows disease progression and improves the quality of life of ALS patients [134].

## 6. Conclusions

DMF represents a promising molecule in the management of many neurodegenerative diseases, thanks to its antioxidant, anti-inflammatory, immunomodulating, and neuroprotective properties. In particular, the study of DMF in the context of CNS disorders could help to better understand not only the effectiveness of the drug but also the underlying mechanisms of action that characterize NDs. Therefore, DMF could represent a new starting point for the management of NDs.

## 7. Future Directions

DMF have found great interest from scientific world in the management of brain pathologies thanks to its neuroprotective effects leading to a reduction of inflammatory and oxidative pathways.

Moreover, DMF is considered a molecule with a secure profile as demonstrated by many studies performed by Gold et al. [135] and Ochi et al. [136]. Considering all of its properties, we are convinced that DMF offer enormous growth potential as a future therapeutic agent for the treatment of NDs (or in association with the general treatment of NDs), which could assist in alleviating symptoms and improving the quality of life of patients.
